# Endoscopic resection of a gastrointestinal stromal tumor using a new mucosal dissection knife in combination with far-view endoscopic submucosal dissection

**DOI:** 10.1055/a-2789-2133

**Published:** 2026-02-17

**Authors:** Nana Hu, Jianguo Zhang

**Affiliations:** 1 Department of Gastroenterology, Aviation General Hospital of China Medical University, Beijing, China

**Fig. 1:**
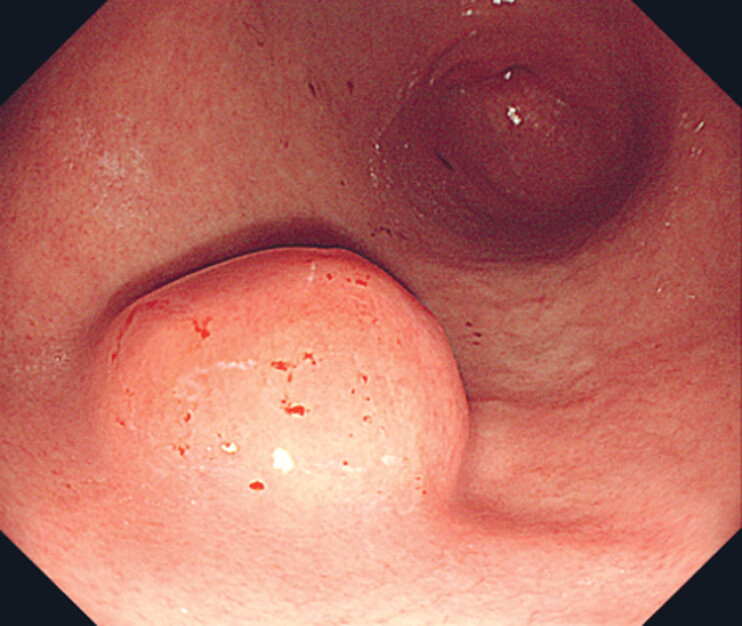
Gastroscopy. A 2.5-cm hemispherical protrusion was found at the greater curvature of the antrum–body junction.

A 59-year-old male patient underwent a gastroscopy at the Aviation General Hospital of China Medical University, which revealed a 2.5 cm hemispherical protrusion at the greater curvature of the antrum–body junction ( Fig. 1 ). A further abdominal enhanced CT scan performed on admission showed an abnormal density at the greater curvature of the antrum–body junction, suggesting a high possibility of gastrointestinal stromal tumors (GISTs).

**Fig. 2:**
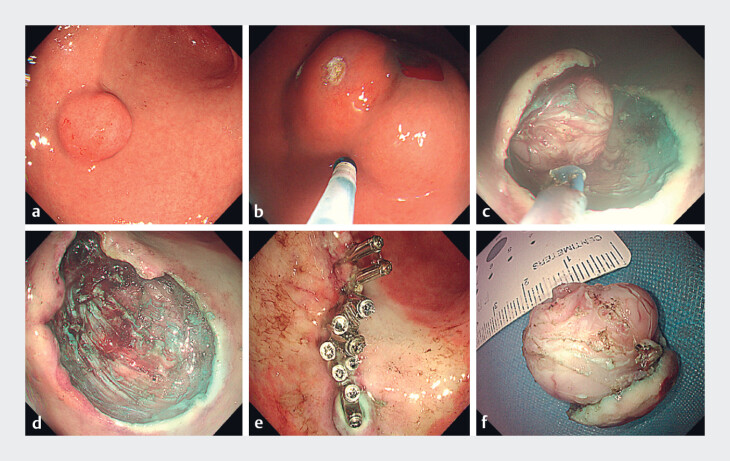
Zhang's knife process. **a** A submucosal tumor measuring approximately 2.1 × 1.8 × 1.5 cm in the greater curvature of the antrum–body junction. **b** Submucosal injection with an endoscopic needle at the oral side of the lesion. **c** “C”-shaped incision in the mucosa using Zhang's knife for FV-ESD, and gradual dissection of the lesion. **d** Post-resection wound. **e** Wound closure with metallic clips. **f** Resected lesion specimen (2.1 × 1.8 × 1.5 cm). FV-ESD, far-view endoscopic submucosal dissection.

**Fig. 3:**
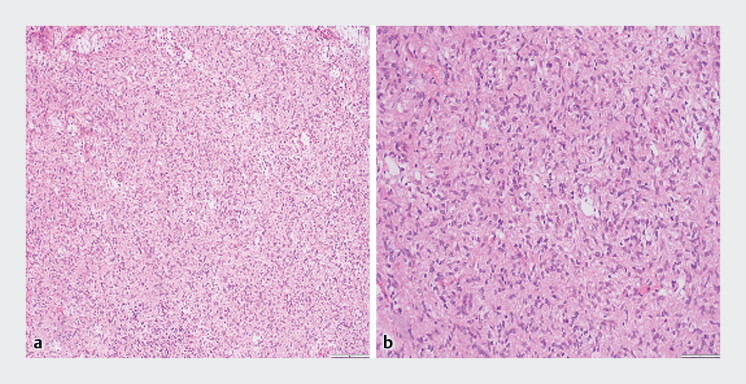
Pathological examination. The tumor cells are spindle-shaped and arranged in fascicles, with mild atypia, and no necrosis is observed.

Therefore, we performed far-view endoscopic submucosal dissection (FV-ESD) using a new mucosal dissection knife. Intraoperatively, a submucosal tumor measuring approximately 2.1 × 1.8 × 1.5 cm was found in the greater curvature of the antrum–body junction( Fig. 2 **a** ). After submucosal injection with an endoscopic needle at the oral side of the lesion ( Fig. 2 **b** ), a “C”-shaped incision was made in the mucosa using a new mucosal dissection knife (KangpaiTe, Beijing, China), and the lesion was gradually dissected ( Fig. 2 **c** ). Finally, the wound was closed with metallic clips ( Fig. 2 **d–f** , Video 1 ). The operation lasted approximately 20 minutes with an estimated blood loss of 10 mL. The patient received supportive treatments and was discharged 4 days later uneventfully. The final histopathology combined with immunophenotype confirms the GIST ( Fig. 3 ).

Procedure of the endoscopic resection of gastrointestinal stromal tumors using Zhang's knife combined with FV-ESD. FV-ESD, far-view endoscopic submucosal dissection.Video 1

ESD has limitations in its application due to its complex operation, long surgical duration, and high risk of complications 1 2 3 4 . A new mucosal dissection knife and the corresponding new technique (FV-ESD) designed can overcome the limitations of traditional ESD knives 5 . FV-ESD obviates a transparent cap, and only the knife tip is inserted to complete the procedure.

This is the first case of using FV-ESD with a new mucosal dissection knife to complete GIST ESD in a simple, fast, and safe way. FV-ESD combined with a new mucosal dissection knife may be an effective method for treating GISTs and is worthy of further study and summary.

Endoscopy_UCTN_Code_TTT_1AO_2AG_3AD
